# Employment and Work Ability of Persons With Brain Tumors: A Systematic Review

**DOI:** 10.3389/fnhum.2020.571191

**Published:** 2020-10-29

**Authors:** Fabiola Silvaggi, Matilde Leonardi, Alberto Raggi, Michela Eigenmann, Arianna Mariniello, Antonio Silvani, Elena Lamperti, Silvia Schiavolin

**Affiliations:** ^1^Unità Operativa Complessa Neurologia, Salute Pubblica, Disabilità, Fondazione Istituti di Ricovero e Cura a Carattere Scientifico Istituto Neurologico Carlo Besta, Milan, Italy; ^2^Unità Operativa Complessa Neurologia 2 – Neuro-Oncologia Clinica, Fondazione Istituti di Ricovero e Cura a Carattere Scientifico Istituto Neurologico Carlo Besta, Milan, Italy

**Keywords:** brain tumors, employment, work ability, public health, RTW

## Abstract

Brain tumors (BT) are between the eight most common cancers among persons aged 40 years, with an average survival time of 10 years for patients affected by non-malignant brain tumor. Some patients continue to work, reporting difficulties in work-related activities, or even job loss. The purpose of the present study was to review the existing information about the ability people with BT to return to work and to identify factors associated with job loss. We performed a systematic review on SCOPUS and EMBASE for peer-reviewed papers that reported studies assessing work ability in patients with BT that were published in the period from January 2010 to January 2020. Out of 800 identified records, 7 articles were selected for analysis, in which 1,507 participants with BT were enrolled overall. Three main themes emerged: the impact of neuropsychological functioning on work productivity, the change of employment status for long-term survivors and issues related to return to work processes. Based on the results of selected studies, it can be concluded that the impact of BT on workforce participation is determined by depressive symptoms and cognitive deficits, as well as by high short-term mortality but also on environmental barriers. Vocational Rehabilitation programs should be implemented to help patients wishing to return to or maintain their current work, as much as possible.

## Introduction

Primary central nervous system (CNS) tumors begin in the brain or spinal cord. Brain and other CNS tumors had an average annual age-adjusted incidence of 11.20 per 100,000 population in the age 15–39 years and 44.47 per 100,000 population in the age 40+ years (Palmer, 2008; Ostrom et al., 2018).

According to World Health Organization Classification of Tumors, the CNS tumors are defined on the basis of the concept of “integrated diagnoses” that is a combination of both phenotypic and genotypic parameters (Louis et al., 2016).

Meningiomas and gliomas are the most common adult brain tumors (BT) accounting for 36 and 24% respectively (Bondy et al., 2008). Approximately one-half of gliomas are glioblastomas, the most frequent malignant primary BT in adults (Ahmed et al., 2014). Survival varies by histology: over 90% of patients with meningioma have a 10 years survival whereas only 5% of patients with malignant BT reach 5 years survival (Pertz et al., 2020).

Treatments' decisions are based on tumor type, location, potential malignancy, severity grading and patient's age and physical conditions. Treatment may require only surveillance but commonly includes surgery, radiotherapy, chemotherapy, or a combination of them, in particular for gliomas (Lawrie et al., 2019).

Most patients with BT present neurocognitive impairments during the trajectory of their disease (Petersen et al., 2014). The degree of cognitive impairment varies from mild to severe across patients' populations. Patients with a WHO grade I–III glioma or meningioma have to cope with potential impairments of socio-cognitive functions for many years and even decades (Louis et al., 2007).

These symptoms cause difficulties in work-related activities, or even job loss. Some patients with BT continue to work, reporting consistent limitations at work due to their health (Ilmarinen, 2008) such as impairment in physical health, working memory and attention (Gallasch et al., 2017). These limitations produce difficulties in the management of job demands. The balance between job demands and employees' resources is known as “work ability,” and is determined by professional knowledge and skills, values, attitudes, motivation and features of work itself (Ilmarinen, 2005). Depressive symptoms, fatigue, cognitive deficits, and difficulties with problem solving and with orientation are among the consequences of BT on workers' ability to perform job duties (Feuerstein et al., 2007).

Past studies with small samples of BT survivors indicated that after 2 years from surgery between 58 and 73% of them were working (Kleinberg et al., 1993; Yabroff et al., 2007).

Patients often consider returning to work (RTW) as a mark of complete recovery (Giovagnoli, 1999) and of regaining a normal life (Spelten et al., 2002). Some reviews focused on the association between recover from cancer and return to work (Steiner et al., 2004; Kennedy et al., 2007; Taskila and Lindbohm, 2007). Consistent use of standardized instruments to assess some of the components of return to work (physical or mental fatigue intensity, role, content, and economic status) would facilitate comparisons of the impact of different cancer sites and treatment settings on the return to a normal work life (Endicott and Nee, 1997; Short et al., 2005).

Others studies analyzed the influence of cancer and its treatment on work performance for those working during treatment or returning to work following treatment (Spelten et al., 2003).

Parallel to this, the main symptom among patients with BT, particularly those with high-grade gliomas, which can affect work ability is fatigue (Bower et al., 2006; Eriksen, 2006; Asher et al., 2016). In the majority of studies, one-quarter to one-third of patients report persistent fatigue for up to 10 years after a cancer diagnosis (Curt et al., 2000; Servaes et al., 2006). The “Cancer related fatigue (CRF)” is associated to somatic and emotional manifestations, such as generalized weakness, diminished concentration or attention, decreased motivation or interest to engage in usual activities, and symptoms of depression and anxiety (Bower, 2014).

In sum, literature findings show that cognitive impairment and fatigue in work-related activities are the most relevant symptoms for BT patients.

The scarcity of information systematically reported about work-related issues for BT patients represents the main gap requiring further investigations (Brown and Kroenke, 2009).

Bridging this gap and evaluating also the impact of environmental factors, is important to design interventions to avoid disability in employment and facilitate maintenance or RTW.

The aim of this systematic review is to examine what is currently known about the work ability of BT survivors and to identify the factors associated to RTW or job loss.

## Methods

### Search Strategy

We performed a comprehensive search on SCOPUS and EMBASE to identify recent primary research papers reporting either observational studies, cross-sectional or longitudinal studies

These designs allowed us to compare critical quantitative data and long- term information on different variables that impact on work ability in workers with brain cancer or to define the factors associated to job loss.

The search covered the period January 2010–January 2020 to find recent data on labor market and the medical treatments for BT patients. The following combinations of key-words were searched within the titles, abstracts, or key-words: (“brain tumor^*^” OR “brain cancer^*^” OR “brain neoplasm^*^” OR glioma OR glioblastoma OR meningioma OR neurinoma OR chordoma OR neurosurg^*^”) OR (“work performance” OR “work engagement” OR “work capacity” OR “capacity to work” OR “work ability” OR “workability” OR “work-ability” OR “ability to work” OR “able to work” OR “unable to work” OR employ^*^ OR unemploy^*^ OR job). Please see Supplementary Materials for the detailed search strategy.

### Articles' Exclusion and Inclusion Criteria

The inclusion criteria were the following: (1) observational studies, cross sectional or longitudinal studies published in English and with an abstract on BT patients in working age; (2) information on the BT patients' work ability after surgery or treatment (defined as the mental and physical capability of performing the job role); (3) no limits in the length of return to work and work ability assessment measured by clinicians or self-reported; (4) information on the factors associated to disease-specific variables (site, stage, treatment, comorbidity), and social and environmental elements that cause loss job. We excluded reviews, commentaries letters to the editors, editorials and case reports. Finally, studies that were based upon caregivers' reports only were excluded as well.

### Paper Selection and Data Extraction

Abstracts of papers were screened by three researchers (FS, AM, ME). To ensure quality and consistency of data extraction, 20% of the abstracts and of full-texts were randomly selected for a second check by a senior researcher (SS) who was blind to the decision of the first one. We determined the overall agreement rate between researchers: if it was below 70%, each of the double-checked abstracts or manuscripts was re-reviewed again by the two researchers to get to a final decision by consensus, and an additional 20% set of abstracts and full-texts was double-checked again.

Extracted information included health conditions and the main characteristics of sample, specifically size, gender distribution, age, percentage of employed subjects for each study.

As we had foreseen, little literature was available on this topic, therefore we did not organize the main contents on work ability into overarching categories. Rather, we described literature findings consistently with the way in which results were reported in each paper. PRISMA statements were made following of specific guidelines (Liberati et al., 2009).

## Results

**Figure 1:**
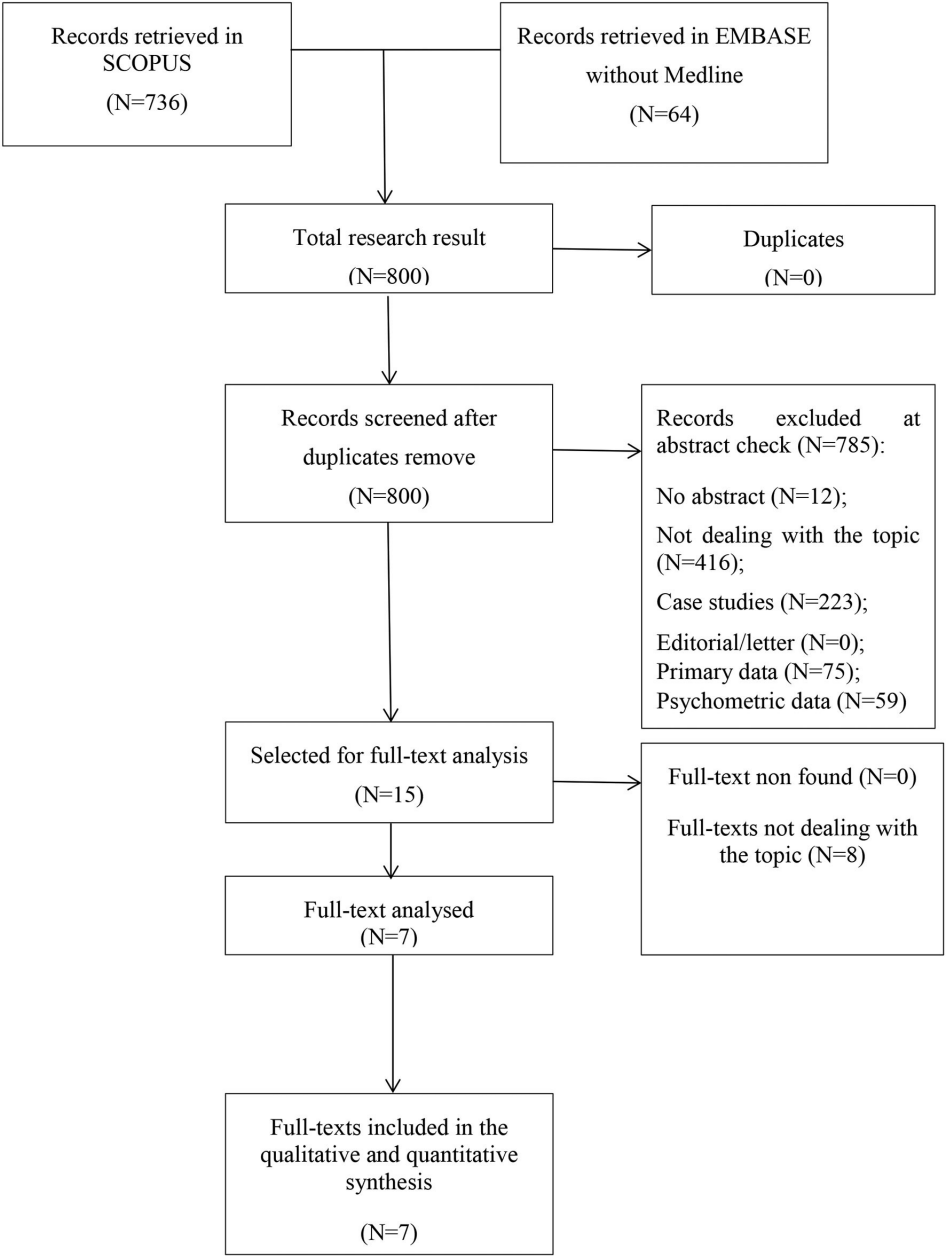
Flowchart of papers' selection.

The initial search returned 800 records. Following abstract screening and full text assessment, seven publications were selected for inclusion in this systematic review (Collins et al., 2013; Rusbridge et al., 2013; Al-Shudifat et al., 2014; Nugent et al., 2014; Pentsova et al., 2016; Starnoni et al., 2018; Tibæk et al., 2018). The rate of agreement between reviewers was 98% at the abstract check and 100% at full-text evaluation. Figure 1 shows the flow diagram of our search process and Table 1 presents summary findings of the publications included in this systematic review.

**Table 1:** Main characteristics and main outcomes of the included studies.

**ID**	**Diagnosis type**	**Sample size**	**Age**	**Men Gender (%)**	**Employed (%)**	**Main results**
Nugent et al. (2014)	Brain tumor	45	43,4	17 (37.8%)	43 (95.5%)	Higher levels of depressive symptoms are associated to a decline in the work ability
Collins et al. (2013)	Brain tumor	233	40,9	79 (33.9%)	209 (89.6%)	Work tasks that involved working memory are more likely to be reported as problematic
Pentsova et al. (2016)	Anaplastic oligoastrocytoma	195	39	111 (57%)	53 (83%)	The functional assessment of patient carried out after surgery impact on change in employment status in future years
Rusbridge et al. (2013)	Brain tumor	34	46	6.66 (19.59%)		VR service improve significantly the work status of brain tumor survivors
Starnoni et al. (2018)	Glioblastoma	125	48,2	77 (61.6%)	112 (89.6%)	Diagnosis and treatment-related symptoms represented the main factors that prevented the resumption of work
Tibæk et al. (2018)	Brain tumor	480	24,4			High mortality and higher levels of impairments reduce the likely of RTW
Al-Shudifat et al. (2014)	Vestibular Schwannomas	395	59,2	188 (48%)	207 (52.4%)	Women and patients above 50 years with dimension cancer >25 mm have a high likely to reduce Work Capacity after surgical treatment and to have major difficulty to return to work

*VR, Vocational Rehabilitation; RTW, Return to work*.

Across the studies, 1.507 participants with a diagnosis of brain cancer were enrolled with average age of 43 years—four studies involved samples of patients with unspecified BT, one with glioblastoma, one with anaplastic oligoastrocytoma and one with vestibular schwannomas.

Two studies evaluated the impact of neuropsychological function on work ability (Collins et al., 2013; Nugent et al., 2014), two studies prospectively addressed employment status change (Rusbridge et al., 2013; Pentsova et al., 2016), three studies addressed the RTW process (Al-Shudifat et al., 2014; Starnoni et al., 2018; Tibæk et al., 2018).

### The Impact of Neuropsychological Functions on Work Productivity

Two studies addressed the work productivity. Nugent et al. performed a prospective longitudinal study in persons with BT that showed how mental attention, flexibility, learning and memory, as well as higher depressive symptoms, were consistently correlated with a decline in the ability to meet occupational demands (Nugent et al., 2014).

The second study (Collins et al., 2013) showed that the problematic work tasks for BT survivors are those involving the cognitive functions such as the flow of events, remembering information, and putting together materials for a task. Among these, work memory is the cognitive function mostly related to difficulties in performing work tasks.

### Changes in Employment Status for Long-Term Survivors

Two studies addressed changes in employment status. Pentsova et al. performed a retrospective study of patients with a diagnosis of anaplastic glioma at Memorial Sloan Kettering Cancer Center from 1999 to 2005 to evaluate employment status of long-term survivors. The results showed that after 5 years or more from diagnosis, 27% previously employed patients suffered from further disease progression and, consequently, prominent neurologic deterioration that did not allow them to work (Pentsova et al., 2016).

The second study (Rusbridge et al., 2013) identified the outcome of a vocational rehabilitation (VR) programme for patients with BT who received several VR sessions between July 2010 and August 2011. After 5 years VR interventions, the results showed that receiving a VR programme had increased employment in patients who did not work at baseline. The VR programme is useful to improve their employment status and to suggest specific job and workload adaptations.

### Return to Work Process

Three studies addressed the RTW process. Starnoni et al. conducted a retrospective study on working—age patients treated with surgery and radiochemotherapy between 2012 and 2015 with the aim to show the incidence of patients resuming their employment and factors related to work capacity. Data showed that glioblastoma diagnosis and treatment have a significant socio-professional impact on being able to continue or resume the previous employment. The main factors involved in not returning to work were related to presence of treatment-related symptoms (36.2%) and disease-related symptoms or neurological deficits (27.5%) (Starnoni et al., 2018).

The second study (Tibæk et al., 2018) showed that, after 1 year, only 27% (15) of patients with BT were able to return to work due to high levels of impairments and to the high incidence of mortality of the disease.

The third study (Al-Shudifat et al., 2014) showed that 79% of patients <64 years returned back to work after surgery within a variable time frame (about 5 months). Among these patients, the women with age > 50 years and tumor diametric > 25 mm had more difficulty in getting back to work.

## Discussion

Findings from this systematic review revealed that patients with BT have an increased likelihood to leave their job due to the impact of neuropsychological symptoms on the ability to work that influences the employment status. The neuropsychological symptoms that have been reported as reducing patients' ability to work are mostly related to depressive symptoms and cognitive deficits.

Some studies reported that depressive symptoms are frequent in patients with BT (Lucchiari et al., 2015; Pranckeviciene and Bunevicius, 2015). It is estimated that 21.7% of BT patients suffers of depression or depressive symptoms related to tumor grade and social support (Huang et al., 2017) and associated to a worse health related quality of life (HRQoL) (Pelletier et al., 2002; McCarty et al., 2017). The impact of financial distress and future uncertainty in brain tumor patients treated with surgery affects HRQoL levels (Kim et al., 2016).

This leads to a higher incidence of suicide within the first year following their diagnosis, to medical complications and low life expectancy (Litofsky et al., 2004; Mainio et al., 2005; Liu et al., 2009; Leistner et al., 2015; Hickmann et al., 2016; Saad et al., 2020).

Moreover, it has been found that these depressive symptoms can be related to unemployment in people with BT (Collins et al., 2013). The role of depression symptoms in patients with BT should be well-understood and studied to develop work place interventions. Unfortunately, some studies showed that there is no agreement on which is the best standardized scale for assessing depression or depressive symptoms in BT patients (Kerr and Kerr, 2001; Williams et al., 2002).

Parallel to this, it has been also showed that unemployment and early retirement in patients with BT is influenced by the duration of disease and by treatment with radiation and chemotherapy (Correa et al., 2008; Collins et al., 2013). Both contribute to a mild decrease in non-verbal recall and in some aspects of executive functions with an early exit from the labor market (Imperato et al., 1990).

On the other hand, some patients can return or maintain their work thanks to the VR services, as shown in one study (Rusbridge et al., 2013). VR services worldwide are currently limited (Omuro et al., 2014), although they may support patients with BT in maintaining their income, identity, and social relationships and improving quality of life. It is very important for companies (especially small- and medium-sized ones) to establish and improve their RTW support systems, with the knowledge that the timing of RTW is variable and might depend on several individual factors (Penedo and Dahn, 2005; Endo et al., 2016). Occupational health professionals may better support BT survivors for RTW dealing with disease-specific variables (site, stage, treatment, comorbidity), and social and environmental elements (Thurin et al., 2019).

Previous studies highlighted the impact of patients' cognitive and emotional problems on work, indicating the need to address these difficulties in VR programs avoiding the loss of skilled personnel within the enterprises (Sherer et al., 1997). VR services can monitor the changes in depressive symptoms and in cognitive and motor deficits even several years after BT diagnosis (Feuerstein et al., 2007).

Evidence on the impact of VR programs on RTW have been found also for patients with traumatic brain injury (TBI) (Matérne et al., 2017). For these patients, the main factor for successful RTW is an individually adapted VR process based on patients with lesser levels of brain damage (mild TBI). The focus of the rehabilitation should be individual and the adaptations to work have to be made constantly (Häggström and Lund, 2008). Also, the social interaction with work colleagues is important, as it provides satisfaction in life (Corrigan et al., 2001).

It is our opinion that VR services can help patients continue working and extend their professional lives as much as possible. These services can be improved with innovations in treatment and with clinical services aimed at a better management of symptoms, rehabilitation and accommodation of disabilities (Spelten et al., 2003). Social and employment policies should be better tailored to support both employers and BT survivors in the RTW process.

Future studies should take into account high mortality rates in the short term of BTs, the differences between self-employed vs. employees, the difference in working in small medium or large enterprises, and the impact of cognitive deficits on work-related activities. It would be also important to investigate which symptoms are connected to job loss across different kind of BTs so as to suggest more tailored interventions or improvements in RTW programs. Future studies should also emphasize the role of environmental factors at policies, systems or services levels that might play a key role on maintaining or returning to work, such as possibility to have smart work or part time work and so on.

Two limitations of this systematic review need to be acknowledged. First, even though our search was quite extensive, we cannot be sure that all relevant articles were found. Second, the presence of heterogeneous samples in diagnosis did not allow a comparison between the studies and did not allow us to analyze in depth the time of RTW or loss of work after surgery. For this reason, a formal meta-analysis was not possible. Only a qualitative synthesis of studies was performed considering each study mostly individually.

## Conclusions

This systematic review indicates the paucity of literature addressing factors associated to RTW or to job loss in BT survivors. Such a lack calls for actions, as the knowledge about work-related issues in BT survivors can help occupational health services to support full recovery and maintenance of employment.

Keeping in mind the small amount of studies found, we can cautiously conclude that the diagnosis of BT influences the workforce participation. In malignant gliomas the survival is still of few years and thus the possibility to continue working, maintain, or RTW, becomes more is difficult. In patient with benign BT the impact of cognitive deficits, fatigue and of treatments' effects often creates work limitations.

However, it was found in one study that VR programs may act supporting patients wishing to return to or maintain their current work. Such services should be available for patients with all types of BT and undergoing different types of treatments so as to keep on participating in the labor workforce. It is also important to underline that VR is not only patient-oriented but also workplace-oriented to allow the highest possible participation in employment if people wish to do it.

## Data Availability Statement

All datasets generated for this study are included in the article/Supplementary Material.

## Author Contributions

FS and SS developed the idea for the review and drafted the manuscript. AR crafted and conducted the bibliographic searches. FS, ME, and AM screened articles for inclusion and extracted data. ML, AR, ME, AM, AS, and EL revised critically for important intellectual content and approved the manuscript. All authors contributed to the article and approved the submitted version.

## Conflict of Interest

The authors declare that the research was conducted in the absence of any commercial or financial relationships that could be construed as a potential conflict of interest.
