# Supplementary material for: Employment and Work Ability of Persons With Brain Tumors: A Systematic Review
Source: Front Hum Neurosci. 2020 Oct 29;14:571191. doi: 10.3389/fnhum.2020.571191 (PMC7658191; doi:10.3389/fnhum.2020.571191)
Supplement: Supplementary file 1 [file Table_1.DOCX]

Supplementary Material

# Supplementary Data.

# Search strategy (performed on January 27, 2020)

SCOPUS

# (TITLE-ABS-KEY ( "brain tumor*" OR "brain cancer*" OR "brain neoplasm*" OR glioma OR glioblastoma OR meningioma OR neurinoma OR chordoma OR neurosurg* ) AND TITLE-ABS-KEY ( "work performance" OR "work engagement" OR "work capacity" OR "capacity to work" OR "work ability" OR "workability" OR "work-ability" OR "ability to work" OR "able to work" OR "unable to work" OR "employ*" OR "unemploy*" OR job ) ) AND PUBYEAR > 2010 AND PUBYEAR < 2020 AND ( LIMIT-TO ( DOCTYPE , "ar" ) ) AND ( EXCLUDE ( SUBJAREA , "BIOC" ) OR EXCLUDE ( SUBJAREA , "ENGI" ) OR EXCLUDE ( SUBJAREA , "PHAR" ) OR EXCLUDE ( SUBJAREA , "CHEM" ) OR EXCLUDE ( SUBJAREA , "COMP" ) OR EXCLUDE ( SUBJAREA , "MATE" ) OR EXCLUDE ( SUBJAREA , "AGRI" ) OR EXCLUDE ( SUBJAREA , "CENG" ) OR EXCLUDE ( SUBJAREA , "PHYS" ) OR EXCLUDE ( SUBJAREA , "IMMU" ) OR EXCLUDE ( SUBJAREA , "MATH" ) OR EXCLUDE ( SUBJAREA , "ENVI" ) OR EXCLUDE ( SUBJAREA , "ARTS" ) OR EXCLUDE ( SUBJAREA , "DECI" ) OR EXCLUDE ( SUBJAREA , "ENER" ) OR EXCLUDE ( SUBJAREA , "VETE" ) OR EXCLUDE ( SUBJAREA , "DENT" ) ) AND ( EXCLUDE ( LANGUAGE , "Chinese" ) OR EXCLUDE ( LANGUAGE , "Japanese" ) OR EXCLUDE ( LANGUAGE , "Russian" ) OR EXCLUDE ( LANGUAGE , "Spanish" ) OR EXCLUDE ( LANGUAGE , "German" ) OR EXCLUDE ( LANGUAGE , "Czech" ) OR EXCLUDE ( LANGUAGE , "French" ) OR EXCLUDE ( LANGUAGE , "Persian" ) OR EXCLUDE ( LANGUAGE , "Hungarian" ) OR EXCLUDE ( LANGUAGE , "Slovenian" ) OR EXCLUDE ( LANGUAGE , "Korean" ) OR EXCLUDE ( LANGUAGE , "Portuguese" ) OR EXCLUDE ( LANGUAGE , "Slovak" ) ) AND ( EXCLUDE ( EXACTKEYWORD , "Adolescent" ) OR EXCLUDE ( EXACTKEYWORD , "Child" ) OR EXCLUDE ( EXACTKEYWORD , "Human Tissue" ) OR EXCLUDE ( EXACTKEYWORD , "Aged, 80 And Over" ) OR EXCLUDE ( EXACTKEYWORD , "Nonhuman" ) OR EXCLUDE ( EXACTKEYWORD , "Very Elderly" ) OR EXCLUDE ( EXACTKEYWORD , "Preschool Child" ) OR EXCLUDE ( EXACTKEYWORD , "Child, Preschool" ) OR EXCLUDE ( EXACTKEYWORD , "Animals" ) OR EXCLUDE ( EXACTKEYWORD , "School Child" ) OR EXCLUDE ( EXACTKEYWORD , "Animal" ) OR EXCLUDE ( EXACTKEYWORD , "Animal Experiment" ) OR EXCLUDE ( EXACTKEYWORD , "Infant" ) OR EXCLUDE ( EXACTKEYWORD , "Animal Model" ) OR EXCLUDE ( EXACTKEYWORD , "Mouse" ) OR EXCLUDE ( EXACTKEYWORD , "Tumor Cell Line" ) OR EXCLUDE ( EXACTKEYWORD , "Animal Tissue" ) OR EXCLUDE ( EXACTKEYWORD , "Rat" ) OR EXCLUDE ( EXACTKEYWORD , "Animal Cell" ) )

Search strategy (performed on January 27, 2020)

EMBASE

('brain tumor*':ti,ab,kw OR 'brain cancer*':ti,ab,kw OR 'brain neoplasm*':ti,ab,kw OR glioma:ti,ab,kw OR glioblastoma:ti,ab,kw OR meningioma:ti,ab,kw OR neurinoma:ti,ab,kw OR chordoma:ti,ab,kw OR neurosurg*:ti,ab,kw OR 'work performance':ti,ab,kw OR 'work engagement':ti,ab,kw OR 'work capacity':ti,ab,kw OR 'capacity to work':ti,ab,kw OR workability:ti,ab,kw OR 'work ability':ti,ab,kw OR 'ability to work':ti,ab,kw OR 'able to work':ti,ab,kw OR 'unable to work':ti,ab,kw OR employ*:ti,ab,kw OR unemploy*:ti,ab,kw OR job:ti,ab,kw) AND [2010-2020]/py AND [embase]/lim NOT ([embase]/lim AND [medline]/lim) AND ('adverse drug reaction'/dm OR 'brain tumor'/dm OR 'complication'/dm OR 'diseases'/dm OR 'epilepsy'/dm OR 'fatigue'/dm OR 'glioblastoma'/dm OR 'glioma'/dm OR 'headache'/dm OR 'meningioma'/dm OR 'metastasis'/dm OR 'neoplasm'/dm OR 'pain'/dm OR 'side effect'/dm OR 'toxicity'/dm) AND ('astrocytoma'/dm OR 'brain metastasis'/dm OR 'neurologic disease'/dm OR 'oligodendroglioma'/dm OR 'primary tumor'/dm) AND ('brain cancer'/dm OR 'central nervous system tumor'/dm OR 'cerebrovascular accident'/dm OR 'edema'/dm OR 'ependymoma'/dm OR 'intracranial tumor'/dm OR 'medulloblastoma'/dm OR 'necrosis'/dm OR 'radiation necrosis'/dm OR 'relapse'/dm OR 'seizure'/dm OR 'solid malignant neoplasm'/dm) AND ([adult]/lim OR [aged]/lim OR [middle aged]/lim OR [young adult]/lim) AND ('case report'/de OR 'clinical article'/de OR 'clinical study'/de OR 'clinical trial'/de OR 'clinical trial (topic)'/de OR 'cohort analysis'/de OR 'comparative effectiveness'/de OR 'controlled clinical trial'/de OR 'controlled study'/de OR 'cross-sectional study'/de OR 'diagnostic test accuracy study'/de OR 'feasibility study'/de OR 'human'/de OR 'human tissue'/de OR 'in vivo study'/de OR 'interview'/de OR 'major clinical study'/de OR 'medical record review'/de OR 'model'/de) AND ('multicenter study'/de OR 'observational study'/de OR 'phase 1 clinical trial'/de OR 'phase 2 clinical trial'/de OR 'phase 3 clinical trial'/de OR 'pilot study'/de OR 'preclinical study'/de OR 'proportional hazards model'/de OR 'prospective study'/de OR 'questionnaire'/de OR 'randomized controlled trial'/de OR 'retrospective study'/de OR 'sample size'/de OR 'systematic review'/de OR 'validation process'/de) AND ('Article'/it OR 'Article in Press'/it) AND (2010:py OR 2011:py OR 2012:py OR 2013:py OR 2014:py OR 2015:py OR 2016:py OR 2017:py OR 2018:py OR 2019:py)
